# Supplementary material for: Pathogenic Mitochondria DNA Mutations: Current Detection Tools and Interventions
Source: Genes (Basel). 2020 Feb 12;11(2):192. doi: 10.3390/genes11020192 (PMC7074468; doi:10.3390/genes11020192)
Supplement: Supplementary file 1 [file genes-11-00192-s001.pdf]

Table S1 mtDNA Mutations Reported in Common Mitochondrial Diseases

**Mitochondria Disease**	**Gene**	**mtDNA Mutation**	**Cited article**
LS	MT-ATP6, complex 5	m.8993T>C	[1–3]
		**m.8993T>G**	[1,3,4]
		m.8993A>G	[1,5]
		**m.9176T>C**	[6–9]
	MT-ND I, complex I	m.3460G>A	[10]
		m.3697G>A	[11]
		m.3946G>A	[12]
	MT-ND2, complex I I	m.4681T>C	[10]
		m.4833A>G	[13]
	MT-ND3, complex I	m.10134C>A	[14]
		m.10158T>C	[12,15,16]
		**m.10191T>C**	[12,15,17–19]
		m.10197G>A	[20–22]
		m.10254G>A	[23]
	MT-ND4, complex I	m.11240C>T	[24]
		m.11777A>C	[19]
		m.11778G>A	[19]
	MT-ND5, complex I	m.12706T>C	[15,25]
		m.13513G>A	[12,13,19,26–29]
		m.13514T>C	[15]
		m.12338T>C	[13]
	MT-ND6, complex I	m.14439G>A	[12]
		m.14502T>C	[13]
		m.14459G>A	[12,30]
		m.14487T>C	[15,31]
	MT-COXII, complex I	m.8108A>G	[13]
	MT-COXIII, complex I	m.9537 C_{Ins}	[32]
	MT-TL I	m.3243A>G	[19]
LHON	MT-ND I, complex I	m.3376G>A	[33]
		m.3394T>C	[34]
		m.3395A>G	[35]
		**m.3460G>A**	[36,37]
		m.3472T>C	[38]
		m.3634A>G	[39]
		m.3635G>A	[40,41]
		m.3697G>A	[42]
		m.3700G>A	[43]
		m.3733G>A	[44]
		m.3866T>C	[45]
		m.4171C>A	[46]
	MT-ND 2, complex I	m.4640C>A	[47]
	MT-CO3, complex 4	m.9804G>A	[48,49]
	MT-ND 3, complex I	m.10197G>A	[21]
	MT-ND 4, complex I	m.10663T>C	[50]
		**m.11778G>A**	[51,52]

	MT-ND 5, complex I	m.13051G>A	[53]
		m.13094T>C	[54]
		m.12848T>C	[55]
	MT-ND6, complex I	m.14279G>A	[56]
		m.14459G>A	[57–59]
		m.14482C>A	[60]
		m.14482C>G	[61]
		**m.14484A>G**	[62–64]
		m.14495A>G	[65]
		m.14502T>C	[66]
		m.14568C>T	[67]
	tRNA	m.15927G>A	[68]
MERRF	MT-TF	m.611G>A	[69]
	MT-TL1	m.3255G>A	[70]
		m.3291T>C	[71]
	MT-TI	m.4279A>G	[72]
		m.4284G>A	[73]
	MT-TW	m.5521G>A	[74]
	MT-TS1	m.7512T>C	[75]
	MT-TK	m.8296A>G	[76]
		**m. 8344A>G**	[20,57,77–80]
		**m.8356T>C**	[81,82]
		m.8361G>A	[83]
		m.8363G>A	[81,82]
	MT-TH	m.12147G>A	[84,85]
	MT-TL2	m.12300G>A	[86]
	MT-ND 5, Complex I	m.13042A>T	[82,87]
	MT-ND6, Complex I	m.14709T>C	[88]
	MT-TP	m.15967G>A	[89]
MELAS	MT-TF	m.583G>A	[90–92]
	MT-TV	m.1616A>G	[93]
		m.1630A>G	[94]
		m.1642G>A	[95,96]
		m.1644G>A	[94,97,98]
	MT-RNR2	m.3093C>G	[99,100]
	MT-TLI	**m.3243A>G**	[57,77,78,81,101–105]
		m.3244G>A	[106–108]
		m.3252A>G	[92,109]
		m.3256C>T	[104,105,110,111]
		m.3258T>C	[112,113]
		m.3260A>G	[114–117]
		**m.3271T>C**	[57,79,103–105,118]
		m.3291T>C	[92,119–121]
	MT-ND1, Complex I	m.3308T>C	[122]

		m.3376G>A	[33,123]
		m.3380G>A	[124]
		m.3481G>A	[125,126]
		m.3697G>A	[42,127]
		m.3946G>A	[127–129]
		m.3949T>C	[127,128]
		m.3959G>A	[130]
		m.3995A>G	[130]
	MT-T1	m.4290T>C	[131]
		m.4320C>T	[132]
	MT-TQ	m.4332G>A	[133]
	MT-TW	m.5540G>A	[134]
	MT-TN	m.5693T>C	[135]
	MT-TC	m.5814T>C	[136]74
		m.5816A>G	[137,138]
	MT-CO1, Complex 4	m.6597C>A	[139]
	MT-TK	m.8316T>C	[140,141]
	MT-CO3, Complex 4	m.9957T>C	[95,142]
	MT-ND 3, Complex I	m.10197G>A	[20]
	MT-TH	m.12146A>G	[143]
		m.12206C>T	[144]
	MT-TL2	m.12299A>C	[145]
	MT-ND5, Complex I	m.12770A>G	[27,146–148]
		m.13042A>T	[87,104]
		m.13045A>C	[27,146,147]
		m.13046T>C	[149]
		m.13084A>T	[27,150]
		m.13513G>A	[27,95,151]
		m.13514A>G	[152]
		m.13528A>G	[150,153]
		m.13849A>C	[154]
	MT-TE	m.14693A>G	[155]
	MT-ND6,Complex 1	m.14453G>A	[156]
	MT-CYB, Complex 3	m.14787delTTAA	[157]
		m.14864T>C	[158]
		m.15092G>A	[159]
		m.15533A>G	[160]
Pearson Syndrome	424 bp deletion from 8578 -14001		[161]
	5182 bp deletion 10,901–16,082		[162]
	**4977 bp deletion from 8482–13460**		[163]
	2461 bp deletion from 10368 12828		[164]
KSS	**4.9 kb deletion from 8469 to 13447**		[165]
	MT-TLI	m.3243A>G	[166]
CPEO	MT-TM	m.4414T>C	[167]
	MT-TN	m.5667G>A	[168]
	MT-TL2	m.12315G>A	[169]

NARP	MT-ATP6,complex V	m.9127–9128 del	[170]
		**m.8993T>G/C**	[171]
MIDD	tRNA	**m.3243A > G**	[172,173]
	MT-ND1, Complex I	m.3421A > G	[174]
	MT-CO2	m.8241 T > G	[175]

Note: Mutations indicated in bold are the primary mutations in the respective diseases

References

1. Rahman, S.; Blok, R.B.; Dahl, H.-H.M.; Danks, D.M.; Kirby, D.M.; Chow, C.W.; Christodoulou, J.; Thorburn, D.R. Leigh syndrome: Clinical features and biochemical and DNA abnormalities. *Ann. Neurol.* **1996**, *39*, 343–351.

2. Weerasinghe, C.; Bui, B.; Vu, T.; Nguyen, H.; Phung, B.-K.; Nguyen, V.-M.; Pham, V.-A.; Cao, V.-H.; Phan, T.-N. Leigh syndrome T8993C mitochondrial DNA mutation: Heteroplasmy and the first clinical presentation in a Vietnamese family. *Mol. Med. Rep.* **2018**.

3. Marie, S.K.N.; Oba-Shinjo, S.M.; Marques-Dias, M.J.; Rosemberg, S.; Kok, F.; Reed, U.C. The prevalence of mitochondrial DNA mutations in Leigh syndrome in a Brazilian series. *Med. Express* **2014**, *1*.

4. Nagashima, T.; Mori, M.; Katayama, K.; Nunomura, M.; Nishihara, H.; Hiraga, H.; Tanaka, S.; Goto, Y.; Nagashima, K. Adult Leigh syndrome with mitochondrial DNA mutation at 8993. *Acta Neuropathol.* **1999**, *97*, 416–422.

5. Han, J.-Y.; Sung, J.-J.; Park, H.-K.; Yoon, B.-N.; Lee, K.-W. Adult onset Leigh syndrome with mitochondrial DNA 8344 A>G mutation. *J. Clin. Neurosci.* **2014**, *21*, 2009–2011.

6. Ronchi, D.; Bordoni, A.; Cosi, A.; Rizzuti, M.; Fassone, E.; Di Fonzo, A.; Servida, M.; Sciacco, M.; Collotta, M.; Ronzoni, M.; et al. Unusual adult-onset Leigh syndrome presentation due to the mitochondrial m.9176T>C mutation. *Biochem. Biophys. Res. Commun.* **2011**, *412*, 245–248.

7. Wilson, C.J.; Wood, N.W.; Leonard, J. V.; Surtees, R. Mitochondrial DNA Point Mutation T9176C in Leigh Syndrome. *J. Child Neurol.* **2000**, *15*, 830–833.

8. Campos, Y.; Martin, M.A.; Rubio, J.C.; Solana, L.G.; Garcia-Benayas, C.; Terradas, J.L.; Arenas, J. Leigh syndrome associated with the T9176C mutation in the ATPase 6 gene of mitochondrial DNA. *Neurology* **1997**, *49*, 595–597.

9. Makino, M.; Horai, S.; Goto, Y.; Nonaka, I. Confirmation that a T-to-C mutation at 9176 in mitochondrial DNA is an additional candidate mutation for Leigh's syndrome. *Neuromuscul. Disord.* **1998**, *8*, 149–151.

10. Hinttala, R.; Smeets, R.; Moilanen, J.S.; Ugalde, C.; Uusimaa, J.; Smeitink, J.A.M.; Majamaa, K. Analysis of mitochondrial DNA sequences in patients with isolated or combined oxidative phosphorylation system deficiency. *J. Med. Genet.* **2006**, *43*, 881–886.

11. Negishi, Y.; Hattori, A.; Takeshita, E.; Sakai, C.; Ando, N.; Ito, T.; Goto, Y.; Saitoh, S. Homoplasmy of a mitochondrial 3697G>A mutation causes Leigh syndrome. *J. Hum. Genet.* **2014**, *59*, 405–407.

12. Ogawa, E.; Shimura, M.; Fushimi, T.; Tajika, M.; Ichimoto, K.; Matsunaga, A.; Tsuruoka, T.; Ishige, M.; Fuchigami, T.; Yamazaki, T.; et al. Clinical validity of biochemical and molecular analysis in diagnosing Leigh syndrome: a study of 106 Japanese patients. *J. Inherit. Metab. Dis.* **2017**, *40*, 685–693.

13. Ma, Y.-Y.; Wu, T.-F.; Liu, Y.-P.; Wang, Q.; Song, J.-Q.; Li, X.-Y.; Shi, X.-Y.; Zhang, W.-N.; Zhao, M.; Hu, L.-Y.; et al. Genetic and biochemical findings in Chinese children with Leigh syndrome. *J. Clin. Neurosci.* **2013**, *20*, 1591–1594.

14. Miller, D.K.; Menezes, M.J.; Simons, C.; Riley, L.G.; Cooper, S.T.; Grimmond, S.M.; Thorburn, D.R.; Christodoulou, J.; Taft, R.J. Rapid Identification of a Novel Complex I MT-ND3 m.10134C>A Mutation in a Leigh Syndrome Patient. *PLoS One* **2014**, *9*, e104879.

15. Lebon, S.; Chol, M.; Benit, P.; Mugnier, C.; Chretien, D.; Giurgea, I.; Kern, I.; Girardin, E.; Hertz-Pannier, L.; de Lonlay, P.; et al. Recurrent de novo mitochondrial DNA mutations in respiratory chain deficiency. *J. Med. Genet.* **2003**, *40*, 896–9.

16. McFarland, R.; Kirby, D.M.; Fowler, K.J.; Ohtake, A.; Ryan, M.T.; Amor, D.J.; Fletcher, J.M.; Dixon, J.W.; Collins, F.A.; Turnbull, D.M.; et al. De novo mutations in the mitochondrialND3 gene as a cause of infantile mitochondrial encephalopathy and complex I deficiency. *Ann. Neurol.* **2004**, *55*, 58–64.

17. Leshinsky-Silver, E.; Lev, D.; Tzofi-Berman, Z.; Cohen, S.; Saada, A.; Yanoov-Sharav, M.; Gilad, E.; Lerman-Sagie, T. Fulminant neurological deterioration in a neonate with Leigh syndrome due to a maternally transmitted missense mutation in the mitochondrial ND3 gene. *Biochem. Biophys. Res. Commun.* **2005**, *334*, 582–587.

18. Taylor, R.W.; Singh-Kler, R.; Hayes, C.M.; Smith, P.E.M.; Turnbull, D.M. Progressive mitochondrial disease resulting from a novel missense mutation in the mitochondrial DNA ND3 gene. *Ann. Neurol.* **2001**, *50*, 104–107.

19. Yu, X.-L.; Yan, C.-Z.; Ji, K.-Q.; Lin, P.-F.; Xu, X.-B.; Dai, T.-J.; Li, W.; Zhao, Y.-Y. Clinical, Neuroimaging, and Pathological Analyses of 13 Chinese Leigh Syndrome Patients with Mitochondrial DNA Mutations. *Chin. Med. J. (Engl).* **2018**, *131*, 2705–2712.

20. Leng, Y.; Liu, Y.; Fang, X.; Li, Y.; Yu, L.; Yuan, Y.; Wang, Z. The mitochondrial DNA 10197 G > A mutation causes MELAS/Leigh overlap syndrome presenting with acute auditory agnosia. *Mitochondrial DNA* **2015**, *26*, 208–212.

21. Wang, K.; Takahashi, Y.; Gao, Z.-L.; Wang, G.-X.; Chen, X.-W.; Goto, J.; Lou, J.-N.; Tsuji, S. Mitochondrial ND3 as the novel causative gene for Leber hereditary optic neuropathy and dystonia. *Neurogenetics* **2009**, *10*, 337–345.

22. Sarzi, E.; Brown, M.D.; Lebon, S.; Chretien, D.; Munnich, A.; Rotig, A.; Procaccio, V. A novel recurrent mitochondrial DNA mutation inND3 gene is associated with isolated complex I deficiency causing Leigh syndrome and dystonia. *Am. J. Med. Genet. Part A* **2007**, *143A*, 33–41.

23. Leshinsky-Silver, E.; Lev, D.; Malinger, G.; Shapira, D.; Cohen, S.; Lerman-Sagie, T.; Saada, A. Leigh disease presenting in utero due to a novel missense mutation in the mitochondrial DNA–ND3. *Mol. Genet. Metab.* **2010**, *100*, 65–70.

24. Xu, B.; Li, X.; Du, M.; Zhou, C.; Fang, H.; Lyu, J.; Yang, Y. Novel mutation of ND4 gene identified by targeted next-generation sequencing in patient with Leigh syndrome. *J. Hum. Genet.* **2017**, *62*, 291–297.

25. Taylor, R.W.; Morris, A.A.M.; Hutchinson, M.; Turnbull, D.M. Leigh disease associated with a novel mitochondrial DNA ND5 mutation. *Eur. J. Hum. Genet.* **2002**,

10, 141–144.

26. Wang, Z.; Qi, X.K.; Yao, S.; Chen, B.; Luan, X.; Zhang, W.; Han, M.; Yuan, Y. Phenotypic patterns of MELAS/LS overlap syndrome associated with m.13513G>A mutation, and neuropathological findings in one autopsy case. *Neuropathology* **2010**, *30*, 606–614.

27. Shanske, S.; Coku, J.; Lu, J.; Ganesh, J.; Krishna, S.; Tanji, K.; Bonilla, E.; Naini, A.B.; Hirano, M.; DiMauro, S. The G13513A Mutation in the ND5 Gene of Mitochondrial DNA as a Common Cause of MELAS or Leigh Syndrome. *Arch. Neurol.* **2008**, *65*.

28. Sudo, A.; Honzawa, S.; Nonaka, I.; Goto, Y. Leigh syndrome caused by mitochondrial DNA G13513A mutation: frequency and clinical features in Japan. *J. Hum. Genet.* **2004**, *49*, 92–96.

29. Chol, M. The mitochondrial DNA G13513A MELAS mutation in the NADH dehydrogenase 5 gene is a frequent cause of Leigh-like syndrome with isolated complex I deficiency. *J. Med. Genet.* **2003**, *40*, 188–191.

30. Kirby, D.M.; Kahler, S.G.; Freckmann, M.-L.; Reddihough, D.; Thorburn, D.R. Leigh disease caused by the mitochondrial DNA G14459A mutation in unrelated families. *Ann. Neurol.* **2000**, *48*, 102–104.

31. Ugalde, C.; Triepels, R.H.; Coenen, M.J.H.; Van Den Heuvel, L.P.; Smeets, R.; Uusimaa, J.; Briones, P.; Campistol, J.; Majamaa, K.; Smeitink, J.A.M.; et al. Impaired complex I assembly in a Leigh syndrome patient with a novel missense mutation in the ND6 gene. *Ann. Neurol.* **2003**, *54*, 665–669.

32. Tiranti, V. A novel frameshift mutation of the mtDNA COIII gene leads to impaired assembly of cytochrome c oxidase in a patient affected by Leigh-like syndrome. *Hum. Mol. Genet.* **2000**, *9*, 2733–2742.

33. Blakely, E.L.; de Silva, R.; King, A.; Schwarzer, V.; Harrower, T.; Dawidek, G.; Turnbull, D.M.; Taylor, R.W. LHON/MELAS overlap syndrome associated with a mitochondrial MTND1 gene mutation. *Eur. J. Hum. Genet.* **2005**, *13*, 623–627.

34. Liang, M.; Guan, M.; Zhao, F.; Zhou, X.; Yuan, M.; Tong, Y.; Yang, L.; Wei, Q.-P.; Sun, Y.-H.; Lu, F.; et al. Leber's hereditary optic neuropathy is associated with mitochondrial ND1 T3394C mutation. *Biochem. Biophys. Res. Commun.* **2009**, *383*, 286–292.

35. Soldath, P.; Wegener, M.; Sander, B.; Rosenberg, T.; Duno, M.; Wibrand, F.; Vissing, J. Leber hereditary optic neuropathy due to a new ND1 mutation. *Ophthalmic Genet.* **2017**, *38*, 480–485.

36. Huoponen, K.; Vilkki, J.; Aula, P.; Nikoskelainen, E.K.; Savontaus, M.L. A new mtDNA mutation associated with Leber hereditary optic neuroretinopathy. *Am. J. Hum. Genet.* **1991**, *48*, 1147–53.

37. Howell, N. Leber hereditary optic neuropathy: respiratory chain dysfunction and degeneration of the optic nerve. *Vision Res.* **1998**, *38*, 1495–1504.

38. Mart ńez-Romero, Í; Herrero-Mart ń, M.D.; Llobet, L.; Emperador, S.; Mart ń-Navarro, A.; Narberhaus, B.; Ascaso, F.J.; L ópez-Gallardo, E.; Montoya, J.; Ruiz-Pesini, E. New M T-ND1 pathologic mutation for Leber hereditary optic neuropathy.

Clin. Experiment. Ophthalmol. **2014**, *42*, 856–864.

39. Carreño-Gago, L.; Gamez, J.; Cámara, Y.; Alvarez de la Campa, E.; Aller-Alvarez, J.S.; Moncho, D.; Salvado, M.; Galan, A.; de la Cruz, X.; Pinós, T.; et al. Identification and characterization of the novel point mutation m.3634A>G in the mitochondrial MT - ND1 gene associated with LHON syndrome. *Biochim. Biophys. Acta - Mol. Basis Dis.* **2017**, *1863*, 182–187.

40. Yang, J.; Zhu, Y.; Tong, Y.; Chen, L.; Liu, L.; Zhang, Z.; Wang, X.; Huang, D.; Qiu, W.; Zhuang, S.; et al. Confirmation of the mitochondrial ND1 gene mutation G3635A as a primary LHON mutation. *Biochem. Biophys. Res. Commun.* **2009**, *386*, 50–54.

41. Brown, M.D.; Zhadanov, S.; Allen, J.C.; Hosseini, S.; Newman, N.J.; Atamonov, V. V.; Mikhailovskaya, I.E.; Sukernik, R.I.; Wallace, D.C. Novel mtDNA mutations and oxidative phosphorylation dysfunction in Russian LHON families. *Hum. Genet.* **2001**, *109*, 33–39.

42. Spruijt, L.; Smeets, H.J.; Hendrickx, A.; Bettink-Remeijer, M.W.; Maat-Kievit, A.; Schoonderwoerd, K.C.; Sluiter, W.; de Coo, I.F.; Hintzen, R.Q. A MELAS-Associated ND1 Mutation Causing Leber Hereditary Optic Neuropathy and Spastic Dystonia. *Arch. Neurol.* **2007**, *64*, 890.

43. Achilli, A.; Iommarini, L.; Olivieri, A.; Pala, M.; Hooshiar Kashani, B.; Reynier, P.; La Morgia, C.; Valentino, M.L.; Liguori, R.; Pizza, F.; et al. Rare Primary Mitochondrial DNA Mutations and Probable Synergistic Variants in Leber's Hereditary Optic Neuropathy. *PLoS One* **2012**, *7*, e42242.

44. Valentino, M.L.; Barboni, P.; Ghelli, A.; Bucchi, L.; Rengo, C.; Achilli, A.; Torroni, A.; Lugaresi, A.; Lodi, R.; Barbiroli, B.; et al. The ND1 gene of complex I is a mutational hot spot for Leber's hereditary optic neuropathy. *Ann. Neurol.* **2004**, *56*, 631–641.

45. Zhou, X.; Qian, Y.; Zhang, J.; Tong, Y.; Jiang, P.; Liang, M.; Dai, X.; Zhou, H.; Zhao, F.; Ji, Y.; et al. Leber's Hereditary Optic Neuropathy Is Associated with the T3866C Mutation in Mitochondrial ND1 Gene in Three Han Chinese Families. *Investig. Opthalmology Vis. Sci.* **2012**, *53*, 4586.

46. Kim, J.Y.; Hwang, J.-M.; Park, S.S. Mitochondrial DNA C4171A/ND1 is a novel primary causative mutation of Leber's hereditary optic neuropathy with a good prognosis. *Ann. Neurol.* **2002**, *51*, 630–634.

47. Niehusmann, P.; Surges, R.; von Wrede, R.D.; Elger, C.E.; Wellmer, J.; Reimann, J.; Urbach, H.; Vielhaber, S.; Bien, C.G.; Kunz, W.S. Mitochondrial dysfunction due to Leber's hereditary optic neuropathy as a cause of visual loss during assessment for epilepsy surgery. *Epilepsy Behav.* **2011**, *20*, 38–43.

48. Dogulu, C.F.; Kansu, T.; Seyrantepe, V.; Ozguc, M.; Topaloglu, H.; Johns, D.R. Mitochondrial DNA analysis in the Turkish Leber's hereditary optic neuropathy population. *Eye* **2001**, *15*, 183–188.

49. Johns, D.R.; Neufeld, M.J. Cytochrome c Oxidase Mutations in Leber Hereditary Optic Neuropathy. *Biochem. Biophys. Res. Commun.* **1993**, *196*, 810–815.

50. Brown, M.D.; Torroni, A.; Reckord, C.L.; Wallace, D.C. Phylogenetic analysis of Leber's hereditary optic neuropathy mitochondrial DNA's indicates multiple

independent occurrences of the common mutations. *Hum. Mutat.* **1995**, *6*, 311–325.

51. Wallace, D.; Singh, G.; Lott, M.; Hodge, J.; Schurr, T.; Lezza, A.; Elsas, L.; Nikoskelainen, E. Mitochondrial DNA mutation associated with Leber's hereditary optic neuropathy. *Science (80-.).* **1988**, *242*, 1427–1430.

52. Xie, S.; Zhang, J.; Sun, J.; Zhang, M.; Zhao, F.; Wei, Q.-P.; Tong, Y.; Liu, X.; Zhou, X.; Jiang, P.; et al. Mitochondrial haplogroup D4j specific variant m.11696G > a(MT-ND4) may increase the penetrance and expressivity of the LHON-associated m.11778G > a mutation in Chinese pedigrees. *Mitochondrial DNA Part A* **2017**, *28*, 434–441.

53. Dombi, E.; Diot, A.; Morten, K.; Carver, J.; Lodge, T.; Fratter, C.; Ng, Y.S.; Liao, C.; Muir, R.; Blakely, E.L.; et al. The m.13051G>A mitochondrial DNA mutation results in variable neurology and activated mitophagy. *Neurology* **2016**, *86*, 1921–1923.

54. Emperador, S.; Vidal, M.; Hernández-Ainsa, C.; Ruiz-Ruiz, C.; Woods, D.; Morales-Becerra, A.; Arruga, J.; Artuch, R.; López-Gallardo, E.; Bayona-Bafaluy, M.P.; et al. The Decrease in Mitochondrial DNA Mutation Load Parallels Visual Recovery in a Leber Hereditary Optic Neuropathy Patient. *Front. Neurosci.* **2018**, *12*.

55. Mayorov, V.; Biousse, V.; Newman, N.J.; Brown, M.D. The role of the ND5 gene in LHON: Characterization of a new, heteroplasmic LHON mutation. *Ann. Neurol.* **2005**, *58*, 807–811.

56. Zhadanov, S.I.; Atamanov, V. V.; Zhadanov, N.I.; Oleinikov, O. V.; Osipova, L.P.; Schurr, T.G. A novel mtDNA ND6 gene mutation associated with LHON in a Caucasian family. *Biochem. Biophys. Res. Commun.* **2005**, *332*, 1115–1121.

57. Wallace, D.C. Mitochondrial defects in neurodegenerative disease. *Ment. Retard. Dev. Disabil. Res. Rev.* **2001**, *7*, 158–166.

58. Gropman, A.; Chen, T.-J.; Perng, C.-L.; Krasnewich, D.; Chernoff, E.; Tifft, C.; Wong, L.-J.C. Variable clinical manifestation of homoplasmic G14459A mitochondrial DNA mutation. *Am. J. Med. Genet.* **2004**, *124A*, 377–382.

59. Shoffner, J.M.; Brown, M.D.; Stugard, C.; June, A.S.; Pollock, S.; Haas, R.H.; Kaufman, A.; Koontz, D.; Kim, Y.; Graham, J.R.; et al. Leber's hereditary optic neuropathy plus dystonia is caused by a mitochondrial DNA point mutation. *Ann. Neurol.* **1995**, *38*, 163–169.

60. Valentino, M.L.; Avoni, P.; Barboni, P.; Pallotti, F.; Rengo, C.; Torroni, A.; Bellan, M.; Baruzzi, A.; Carelli, V. Mitochondrial DNA nucleotide changes C14482G and C14482A in the ND6 gene are pathogenic for Leber's hereditary optic neuropathy. *Ann. Neurol.* **2002**, *51*, 774–778.

61. Howell, N.; Bogolin, C.; Jamieson, R.; Marenda, D.R.; Mackey, D.A. mtDNA Mutations That Cause Optic Neuropathy: How Do We Know? *Am. J. Hum. Genet.* **1998**, *62*, 196–202.

62. Johns, D.R.; Neufeld, M.J.; Park, R.D. An ND-6 mitochondrial DNA mutation associated with leber hereditary optic neuropathy. *Biochem. Biophys. Res. Commun.* **1992**, *187*, 1551–1557.

63. Howell, N.; Kubacka, I.; Xu, M.; McCullough, D.A. Leber hereditary optic

neuropathy: involvement of the mitochondrial ND1 gene and evidence for an intragenic suppressor mutation. *Am. J. Hum. Genet.* **1991**, *48*, 935–42.

64. Catarino, C.B.; Ahting, U.; Gusic, M.; Iuso, A.; Repp, B.; Peters, K.; Biskup, S.; von Livonius, B.; Prokisch, H.; Klopstock, T. Characterization of a Leber’s hereditary optic neuropathy (LHON) family harboring two primary LHON mutations m.11778G > A and m.14484T > C of the mitochondrial DNA. *Mitochondrion* **2017**, *36*, 15–20.

65. Chinnery, P.F.; Brown, D.T.; Andrews, R.M.; Singh-Kler, R.; Riordan-Eva, P.; Lindley, J.; Applegarth, D.A.; Turnbull, D.M.; Howell, N. The mitochondrial ND6 gene is a hot spot for mutations that cause Leber’s hereditary optic neuropathy. *Brain* **2001**, *124*, 209–218.

66. Zhao, F.; Guan, M.; Zhou, X.; Yuan, M.; Liang, M.; Liu, Q.; Liu, Y.; Zhang, Y.; Yang, L.; Tong, Y.; et al. Leber’s hereditary optic neuropathy is associated with mitochondrial ND6 T14502C mutation. *Biochem. Biophys. Res. Commun.* **2009**, *389*, 466–472.

67. Fauser, S.; Leo-Kottler, B.; Besch, D.; Luberichs, J. Confirmation of the 14568 mutation in the mitochondrial ND6 gene as causative in Leber’s hereditary optic neuropathy. *Ophthalmic Genet.* **2002**, *23*, 191–7.

68. Zhang, J.; Ji, Y.; Liu, X.; Chen, J.; Wang, B.; Zhang, M.; Guan, M.-X. Leber’s hereditary optic neuropathy caused by a mutation in mitochondrial tRNAThr in eight Chinese pedigrees. *Mitochondrion* **2018**, *42*, 84–91.

69. Mancuso, M.; Filosto, M.; Mootha, V.K.; Rocchi, A.; Pistolesi, S.; Murri, L.; DiMauro, S.; Siciliano, G. A novel mitochondrial tRNAPhe mutation causes MERRF syndrome. *Neurology* **2004**, *62*, 2119–2121.

70. Nishigaki, Y.; Tadesse, S.; Bonilla, E.; Shungu, D.; Hersh, S.; Keats, B.J.B.; Berlin, C.I.; Goldberg, M.F.; Vockley, J.; DiMauro, S.; et al. A novel mitochondrial tRNALeu(UUR) mutation in a patient with features of MERRF and Kearns–Sayre syndrome. *Neuromuscul. Disord.* **2003**, *13*, 334–340.

71. Liu, K.; Zhao, H.; Ji, K.; Yan, C. MERRF/MELAS overlap syndrome due to the m.3291T>C mutation. *Metab. Brain Dis.* **2014**, *29*, 139–144.

72. Zsurka, G.; Becker, F.; Heinen, M.; Gdynia, H.-J.; Lerche, H.; Kunz, W.S.; Weber, Y.G. Mutation in the mitochondrial tRNAIle gene causes progressive myoclonus epilepsy. *Seizure* **2013**, *22*, 483–486.

73. Hahn, A.; Sch änzer, A.; Neubauer, B.A.; Gizewski, E.; Ahting, U.; Rolinski, B. MERRF-Like Phenotype Associated with a Rare Mitochondrial tRNAIle Mutation (m.4284 G>A). *Neuropediatrics* **2011**, *42*, 148–151.

74. Silvestri, G.; Rana, M.; DiMuzio, A.; Uncini, A.; Tonali, P.; Servidei, S. A late-onset mitochondrial myopathy is associated with a novel mitochondrial DNA (mtDNA) point mutation in the tRNATrp gene. *Neuromuscul. Disord.* **1998**, *8*, 291–295.

75. Nakamura, M.; Nakano, S.; Goto, Y.; Ozawa, M.; Nagahama, Y.; Fukuyama, H.; Akiguchi, I.; Kaji, R.; Kimura, J. A Novel Point Mutation in the Mitochondrial tRNASer(UCN) Gene Detected in a Family with MERRF/MELAS Overlap Syndrome. *Biochem. Biophys. Res. Commun.* **1995**, *214*, 86–93.

76. Ahadi, A.M.; Sadeghizadeh, M.; Houshmand, M.; Gharagoozli, K.; Banoei, M.M.; Panahai, M.S.S. An A8296G mutation in the MT-TK gene of a patient with epilepsy - A disease-causing mutation or rare polymorphism? *Neurol. Neurochir. Pol.* **2008**.

77. Zapico, S. mtDNA Mutations and Their Role in Aging, Diseases and Forensic Sciences. *Aging Dis.* **2013**, *4*, 364–380.

78. Wallace, D.C. A mitochondrial bioenergetic etiology of disease. *J. Clin. Invest.* **2013**, *123*, 1405–1412.

79. Berardo, A.; Musumeci, O.; Toscano, A. Cardiological manifestations of mitochondrial respiratory chain disorders. *Acta Myol.* 2011.

80. Mancuso, M.; Petrozzi, L.; Filosto, M.; Nesti, C.; Rocchi, A.; Choub, A.; Pistolesi, S.; Massetani, R.; Fontanini, G.; Siciliano, G. MERRF syndrome without ragged-red fibers: The need for molecular diagnosis. *Biochem. Biophys. Res. Commun.* **2007**, *354*, 1058–1060.

81. Kabunga, P.; Lau, A.K.; Phan, K.; Puranik, R.; Liang, C.; Davis, R.L.; Sue, C.M.; Sy, R.W. Systematic review of cardiac electrical disease in Kearns–Sayre syndrome and mitochondrial cytopathy. *Int. J. Cardiol.* **2015**, *181*, 303–310.

82. Lorenzoni, P.J.; Scola, R.H.; Kay, C.S.K.; Silvado, C.E.S.; Werneck, L.C. When should MERRF (myoclonus epilepsy associated with ragged-red fibers) be the diagnosis? *Arq. Neuropsiquiatr.* **2014**, *72*, 803–811.

83. Rossmanith, W.; Raffelsberger, T.; Roka, J.; Kornek, B.; Feucht, M.; Bittner, R.E. The expanding mutational spectrum of MERRF substitution G8361A in the mitochondrial tRNALys gene. *Ann. Neurol.* **2003**, *54*, 820–823.

84. Melone, M.A.B.; Tessa, A.; Petrini, S.; Lus, G.; Sampaolo, S.; di Fede, G.; Santorelli, F.M.; Cotrufo, R. Revelation of a New Mitochondrial DNA Mutation (G12147A) in a MELAS/MERFF Phenotype. *Arch. Neurol.* **2004**, *61*, 269.

85. Taylor, R.W.; Schaefer, A.M.; McDonnell, M.T.; Petty, R.K.H.; Thomas, A.M.; Blakely, E.L.; Hayes, C.M.; McFarland, R.; Turnbull, D.M. Catastrophic presentation of mitochondrial disease due to a mutation in the tRNAHis gene. *Neurology* **2004**, *62*, 1420–1423.

86. Mart ín-Jim énez, R.; Mart ín-Hern ández, E.; Cabello, A.; Garc ía-Silva, M.T.; Arenas, J.; Campos, Y. Clinical and cellular consequences of the mutation m.12300G>A in the mitochondrial tRNALeu(CUN) gene. *Mitochondrion* **2012**, *12*, 288–293.

87. Schinwelski, M.; Kierdaszuk, B.; Dulski, J.; Tońska, K.; Kodroń, A.; Sitek, E.J.; Bartnik, E.; Kamińska, A.; Kwieciński, H.; Sławek, J. Changing phenotypic expression in a patient with a mitochondrial encephalopathy due to 13042G>A de novo mutation—a 5 year follow up. *Metab. Brain Dis.* **2015**, *30*, 1083–1085.

88. Ban, R.; Guo, J.-H.; Pu, C.-Q.; Shi, Q.; Liu, H.-X.; Zhang, Y.-T. A Novel Mutation of Mitochondrial T14709C Causes Myoclonic Epilepsy with Ragged Red Fibers Syndrome in a Chinese Patient. *Chin. Med. J. (Engl).* **2018**, *131*, 1569–1574.

89. Blakely, E.L.; Trip, S.A.; Swalwell, H.; He, L.; Wren, D.R.; Rich, P.; Turnbull, D.M.; Omer, S.E.; Taylor, R.W. A New Mitochondrial Transfer RNAPro Gene Mutation Associated With Myoclonic Epilepsy With Ragged-Red Fibers and Other Neurological Features. *Arch. Neurol.* **2009**, *66*.

90. Darin, N.; Oldfors, A.; Moslemi, A.-R.; Holme, E.; Tulinius, M. The incidence of mitochondrial encephalomyopathies in childhood: Clinical features and morphological, biochemical, and DNA abnormalities. *Ann. Neurol.* **2001**, *49*, 377–383.

91. Hanna, M.G.; Nelson, I.P.; Morgan-Hughes, J.A.; Wood, N.W. MELAS: a new disease associated mitochondrial DNA mutation and evidence for further genetic heterogeneity. *J. Neurol. Neurosurg. Psychiatry* **1998**, *65*, 512–517.

92. Goto, Y.-I. Clinical features of melas and mitochondrial DNA mutations. *Muscle Nerve* **1995**, *18*, S107–S112.

93. Toyoshima, Y.; Tanaka, Y.; Satomi, K. MELAS syndrome associated with a new mitochondrial tRNA-Val gene mutation (m.1616A>G). *BMJ Case Rep.* **2017**, bcr-2017-220934.

94. F., M.; A., B.; M., D.; M., G.; M.G., M. A novel mtDNA point mutation in tRNA(Val) is associated with hypertrophic cardiomyopathy and MELAS. *Ital. Heart J.* **2004**.

95. Taylor, R.W.; Chinnery, P.F.; Haldane, F.; Morris, A.A.M.; Bindoff, L.A.; Turnbull, D.M.; Wilson, J. MELAS associated with a mutation in the valine transfer RNA gene of mitochondrial DNA. *Ann. Neurol.* **1996**, *40*, 459–462.

96. de Coo, I.F.M.; Sistermans, E.A.; de Wijs, I.J.; Catsman-Berrevoets, C.; Busch, H.F.M.; Scholte, H.R.; de Klerk, J.B.C.; van Oost, B.A.; Smeets, H.J.M. A mitochondrial tRNAVal gene mutation (G1642A) in a patient with mitochondrial myopathy, lactic acidosis, and stroke-like episodes. *Neurology* **1998**, *50*, 293–295.

97. Fraidakis, M.J.; Jardel, C.; Allouche, S.; Nelson, I.; Auré, K.; Slama, A.; Lemière, I.; Thenint, J.P.; Hamon, J.B.; Zagnoli, F.; et al. Phenotypic diversity associated with the MT-TV gene m.1644G>A mutation, a matter of quantity. *Mitochondrion* **2014**, *15*, 34–39.

98. Tanji, K.; Kaufmann, P.; Naini, A.B.; Lu, J.; Parsons, T.C.; Wang, D.; Willey, J.Z.; Shanske, S.; Hirano, M.; Bonilla, E.; et al. A novel tRNAVal mitochondrial DNA mutation causing MELAS. *J. Neurol. Sci.* **2008**, *270*, 23–27.

99. Li, J.-Y.; Hsieh, R.-H.; Peng, N.-J.; Lai, P.-H.; Lee, C.-F.; Lo, Y.-K.; Wei, Y.-H. A Follow-up Study in a Taiwanese Family with Mitochondrial Myopathy, Encephalopathy, Lactic Acidosis and Stroke-like Episodes Syndrome. *J. Formos. Med. Assoc.* **2007**, *106*, 528–536.

100. Hsieh, R.-H.; Li, J.-Y.; Pang, C.-Y.; Wei, Y.-H. A Novel Mutation in the Mitochondrial 16S rRNA Gene in a Patient with MELAS Syndrome, Diabetes Mellitus, Hyperthyroidism and Cardiomyopathy. *J. Biomed. Sci.* **2001**, *8*, 328–335.

101. Greaves, L.C.; Reeve, A.K.; Taylor, R.W.; Turnbull, D.M. Mitochondrial DNA and disease. *J. Pathol.* **2012**, *226*, 274–286.

102. Zhu, C.-C.; Traboulsi, E.I.; Parikh, S. Ophthalmological findings in 74 patients with mitochondrial disease. *Ophthalmic Genet.* **2017**, *38*, 67–69.

103. Vydt, T.C.G.; de Coo, R.F.M.; Soliman, O.I.I.; ten Cate, F.J.; van Geuns, R.-J.M.; Vletter, W.B.; Schoonderwoerd, K.; van den Bosch, B.J.C.; Smeets, H.J.M.; Geleijnse, M.L. Cardiac Involvement in Adults With m.3243A>G MELAS Gene Mutation. *Am. J. Cardiol.* **2007**, *99*, 264–269.

104. Lorenzoni, P.J.; Werneck, L.C.; Kay, C.S.K.; Silvado, C.E.S.; Scola, R.H. When should MELAS (Mitochondrial myopathy, Encephalopathy, Lactic Acidosis, and Stroke-like episodes) be the diagnosis? *Arq. Neuropsiquiatr.* **2015**, *73*, 959–967.

105. Wang, Y.-X.; Le, W.-D. Progress in Diagnosing Mitochondrial Myopathy, Encephalopathy, Lactic Acidosis, and Stroke-like Episodes. *Chin. Med. J. (Engl).* **2015**, *128*, 1820–1825.

106. Mimaki, M.; Hatakeyama, H.; Ichiyama, T.; Isumi, H.; Furukawa, S.; Akasaka, M.; Kamei, A.; Komaki, H.; Nishino, I.; Nonaka, I.; et al. Different effects of novel mtDNA G3242A and G3244A base changes adjacent to a common A3243G mutation in patients with mitochondrial disorders. *Mitochondrion* **2009**, *9*, 115–122.

107. Queen, R.A.; Steyn, J.S.; Lord, P.; Elson, J.L. Mitochondrial DNA sequence context in the penetrance of mitochondrial t-RNA mutations: A study across multiple lineages with diagnostic implications. *PLoS One* **2017**, *12*, e0187862.

108. Kirino, Y.; Goto, Y. -i.; Campos, Y.; Arenas, J.; Suzuki, T. Specific correlation between the wobble modification deficiency in mutant tRNAs and the clinical features of a human mitochondrial disease. *Proc. Natl. Acad. Sci.* **2005**, *102*, 7127–7132.

109. Morten, K.J.; Cooper, J.M.; Brown, G.K.; Lake, B.D.; PIke, D.; Poulton, J. A new point mutation associated with mitochondrial encephalomyopathy. *Hum. Mol. Genet.* **1993**, *2*, 2081–2087.

110. Jeppesen, T.D.; Schwartz, M.; Hansen, K.; Danielsen, E.R.; Wibrand, F.; Vissing, J. Late onset of stroke-like episode associated with a 3256C→T point mutation of mitochondrial DNA. *J. Neurol. Sci.* **2003**, *214*, 17–20.

111. Rossmanith, W.; Karwan, R.M. Impairment of tRNA processing by point mutations in mitochondrial tRNA Leu(UUR) associated with mitochondrial diseases. *FEBS Lett.* **1998**, *433*, 269–274.

112. Sternberg, D. Mitochondrial DNA transfer RNA gene sequence variations in patients with mitochondrial disorders. *Brain* **2001**, *124*, 984–994.

113. Campos, Y. Two pathogenic mutations in the mitochondrial DNA tRNA Leu(UUR) gene (T3258C and A3280G) resulting in variable clinical phenotypes. *Neuromuscul. Disord.* **2003**, *13*, 416–420.

114. Mariotti, C.; Tiranti, V.; Carrara, F.; Dallapiccola, B.; DiDonato, S.; Zeviani, M. Defective respiratory capacity and mitochondrial protein synthesis in transformant cybrids harboring the tRNA(Leu(UUR)) mutation associated with maternally inherited myopathy and cardiomyopathy. *J. Clin. Invest.* **1994**, *93*, 1102–1107.

115. Hammans, S.R.; Sweeney, M.G.; Hanna, M.G.; Brockington, M.; Morgan-Hughes, J.A.; Harding, A.E. The mitochondrial DNA transfer RNA Leu (UUR) A→G(3243) mutation: A clinical and genetic study. *Brain* **1995**, *118*, 721–734.

116. Zeviani, M.; Gellera, C.; Antozzi, C.; Rimoldi, M.; Morandi, L.; Tiranti, V.; DiDonato, S.; Villani, F. Maternally inherited myopathy and cardiomyopathy: association with mutation in mitochondrial DNA tRNALeu(UUR). *Lancet* **1991**, *338*, 143–147.

117. Nishino, I.; Komatsu, M.; Kodama, S.; Horai, S.; Nonaka, I.; Goto, Y.-I. The 3260 mutation in mitochondrial DNA can cause mitochondrial myopathy, encephalopathy, lactic acidosis, and strokelike episodes (MELAS). *Muscle Nerve* **1996**, *19*, 1603–1604.

118. Goto, Y.; Nonaka, I.; Horai, S. A mutation in the tRNALeu(UUR) gene associated with the MELAS subgroup of mitochondrial encephalomyopathies. *Nature* **1990**, *348*, 651–653.

119. Goto, Y.I.; Tsugane, K.; Tanabe, Y.; Nonaka, I.; Horai, S. A New Point Mutation at Nucleotide Pair-3291 of the Mitochondrial Transfer-RNALeu(Uur) Gene in a Patient with Mitochondrial Myopathy, Encephalopathy, Lactic-Acidosis, and Stroke-Like Episodes (MELAS). *Biochem. Biophys. Res. Commun.* **1994**, *202*, 1624–1630.

120. Salsano, E.; Giovagnoli, A.R.; Morandi, L.; Maccagnano, C.; Lamantea, E.; Marchesi, C.; Zeviani, M.; Pareyson, D. Mitochondrial dementia: A sporadic case of progressive cognitive and behavioral decline with hearing loss due to the rare m.3291T>C MELAS mutation. *J. Neurol. Sci.* **2011**, *300*, 165–168.

121. Sunami, Y.; Sugaya, K.; Chihara, N.; Goto, Y.; Matsubara, S. Variable phenotypes in a family with mitochondrial encephalomyopathy harboring a 3291T > C mutation in mitochondrial DNA. *Neurol. Sci.* **2011**, *32*, 861–864.

122. Campos, Y.; Martín, M.A.; Rubio, J.C.; Olmo, M.C.G. del; Cabello, A.; Arenas, J. Bilateral Striatal Necrosis and MELAS Associated with a New T3308C Mutation in the Mitochondrial ND1 Gene. *Biochem. Biophys. Res. Commun.* **1997**, *238*, 323–325.

123. Pätsi, J.; Maliniemi, P.; Pakanen, S.; Hinttala, R.; Uusimaa, J.; Majamaa, K.; Nyström, T.; Kervinen, M.; Hassinen, I.E. LHON/MELAS overlap mutation in ND1 subunit of mitochondrial complex I affects ubiquinone binding as revealed by modeling in Escherichia coli NDH-1. *Biochim. Biophys. Acta - Bioenerg.* **2012**, *1817*, 312–318.

124. Horváth, R.; Reilmann, R.; Holinski-Feder, E.; Ringelstein, E.B.; Klopstock, T. The role of complex I genes in MELAS: A novel heteroplasmic mutation 3380G>A in ND1 of mtDNA. *Neuromuscul. Disord.* **2008**, *18*, 553–556.

125. Malfatti, E.; Bugiani, M.; Invernizzi, F.; de Souza, C.F.-M.; Farina, L.; Carrara, F.; Lamantea, E.; Antozzi, C.; Confalonieri, P.; Sanseverino, M.T.; et al. Novel mutations of ND genes in complex I deficiency associated with mitochondrial encephalopathy. *Brain* **2007**, *130*, 1894–1904.

126. Moslemi, A.-R.; Darin, N.; Tulinius, M.; Wiklund, L.-M.; Holme, E.; Oldfors, A. Progressive Encephalopathy and Complex I Deficiency Associated with Mutations in MTND1. *Neuropediatrics* **2008**, *39*, 24–28.

127. Kirby, D.M. Mutations of the mitochondrial ND1 gene as a cause of MELAS. *J. Med. Genet.* **2004**, *41*, 784–789.

128. Swalwell, H.; Kirby, D.M.; Blakely, E.L.; Mitchell, A.; Salemi, R.; Sugiana, C.; Compton, A.G.; Tucker, E.J.; Ke, B.-X.; Lamont, P.J.; et al. Respiratory chain complex I deficiency caused by mitochondrial DNA mutations. *Eur. J. Hum. Genet.* **2011**, *19*, 769–775.

129. Kervinen, M.; Hinttala, R.; Helander, H.M.; Kurki, S.; Uusimaa, J.; Finel, M.; Majamaa, K.; Hassinen, I.E. The MELAS mutations 3946 and 3949 perturb the critical structure in a conserved loop of the ND1 subunit of mitochondrial complex I. *Hum. Mol. Genet.* **2006**, *15*, 2543–2552.

130. Lin, J.; Zhao, C.-B.; Lu, J.-H.; Wang, H.-J.; Zhu, W.-H.; Xi, J.-Y.; Lu, J.; Luo, S.-S.; Ma, D.; Wang, Y.; et al. Novel mutations m.3959G>A and m.3995A>G in

mitochondrial gene MT-ND1 associated with MELAS. *Mitochondrial DNA* **2014**, *25*, 56–62.

131. Limongelli, A. Variable penetrance of a familial progressive necrotising encephalopathy due to a novel tRNAIle homoplasmic mutation in the mitochondrial genome. *J. Med. Genet.* **2004**, *41*, 342–349.

132. Santorelli, F.M.; Mak, S.C.; Vazquezacevedo, M.; Gonzalezastiazaran, A.; Ridaurasanz, C.; Gonzalezhalphen, D.; Dimauro, S. A Novel Mitochondrial DNA Point Mutation Associated with Mitochondrial Encephalocardiomyopathy. *Biochem. Biophys. Res. Commun.* **1995**, *216*, 835–840.

133. Bataillard, M.; Chatzoglou, E.; Rumbach, L.; Sternberg, D.; Tournade, A.; Laforet, P.; Jardel, C.; Maisonobe, T.; Lombes, A. Atypical MELAS syndrome associated with a new mitochondrial tRNA glutamine point mutation. *Neurology* **2001**, *56*, 405–407.

134. Cohen, B.H. MERRF. In *Mitochondrial Case Studies*; Elsevier, 2016; pp. 31–36 ISBN 9780128011492.

135. Coulbault, L.; Herlicoviez, D.; Chapon, F.; Read, M.-H.; Penniello, M.-J.; Reynier, P.; Fayet, G.; Lombès, A.; Jauzac, P.; Allouche, S. A novel mutation in the mitochondrial tRNAAsn gene associated with a lethal disease. *Biochem. Biophys. Res. Commun.* **2005**, *329*, 1152–1154.

136. Sternberg, D.; Danan, C.; Lombes, A.; Laforet, P.; Girodon, E.; Goossens, M.; Amselem, S. Exhaustive Scanning Approach to Screen All the Mitochondrial tRNA Genes for Mutations and Its Application to the Investigation of 35 Independent Patients with Mitochondrial Disorders. *Hum. Mol. Genet.* **1998**, *7*, 33–42.

137. McFarland, R.; Chinnery, P.F.; Blakely, E.L.; Schaefer, A.M.; Morris, A.A.M.; Foster, S.M.; Tuppen, H.A.L.; Ramesh, V.; Dorman, P.J.; Turnbull, D.M.; et al. Homoplasmy, heteroplasmy, and mitochondrial dystonia. *Neurology* **2007**, *69*, 911–916.

138. Schaefer, A.M.; McFarland, R.; Blakely, E.L.; He, L.; Whittaker, R.G.; Taylor, R.W.; Chinnery, P.F.; Turnbull, D.M. Prevalence of mitochondrial DNA disease in adults. *Ann. Neurol.* **2008**, *63*, 35–39.

139. Lamperti, C.; Diodato, D.; Lamantea, E.; Carrara, F.; Ghezzi, D.; Mereghetti, P.; Rizzi, R.; Zeviani, M. MELAS-like encephalomyopathy caused by a new pathogenic mutation in the mitochondrial DNA encoded cytochrome c oxidase subunit I. *Neuromuscul. Disord.* **2012**, *22*, 990–994.

140. Campos, Y.; Lorenzo, G.; Martín, M.A.; Torregrosa, A.; del Hoyo, P.; Rubio, J.C.; García, A.; Arenas, J. A mitochondrial tRNALys gene mutation (T8316C) in a patient with mitochondrial myopathy, lactic acidosis, and stroke-like episodes. *Neuromuscul. Disord.* **2000**, *10*, 493–496.

141. SISSLER, M. Aminoacylation properties of pathology-related human mitochondrial tRNALys variants. *RNA* **2004**, *10*, 841–853.

142. Manfredi, G.; Schon, E.A.; Moraes, C.T.; Bonilla, E.; Berry, G.T.; Sladky, J.T.; Dimauro, S. A new mutation associated with MELAS is located in a mitochondrial DNA polypeptide-coding gene. *Neuromuscul. Disord.* **1995**, *5*, 391–398.

143. Calvaruso, M.A.; Willemsen, M.A.; Rodenburg, R.J.; van den Brand, M.; Smeitink, J.A.M.; Nijtmans, L. New mitochondrial tRNAHIS mutation in a family with lactic

acidosis and stroke-like episodes (MELAS). *Mitochondrion* **2011**, *11*, 778–782.

144. Blakely, E.L.; Yarham, J.W.; Alston, C.L.; Craig, K.; Poulton, J.; Brierley, C.; Park, S.-M.; Dean, A.; Xuereb, J.H.; Anderson, K.N.; et al. Pathogenic Mitochondrial tRNA Point Mutations: Nine Novel Mutations Affirm Their Importance as a Cause of Mitochondrial Disease. *Hum. Mutat.* **2013**, *34*, 1260–1268.

145. Abu-Amero, K.K.; Ozand, P.T.; Al-Dhalaan, H. Novel Mitochondrial DNA Transversion Mutation in Transfer Ribonucleic Acid for Leucine 2 (CUN) in a Patient With the Clinical Features of MELAS. *J. Child Neurol.* **2006**, *21*, 971–972.

146. Liolitsa, D.; Rahman, S.; Benton, S.; Carr, L.J.; Hanna, M.G. Is the mitochondrial complex I ND5 gene a hot-spot for MELAS causing mutations? *Ann. Neurol.* **2003**, *53*, 128–132.

147. Pereira, L.; Soares, P.; Radivojac, P.; Li, B.; Samuels, D.C. Comparing Phylogeny and the Predicted Pathogenicity of Protein Variations Reveals Equal Purifying Selection across the Global Human mtDNA Diversity. *Am. J. Hum. Genet.* **2011**, *88*, 433–439.

148. Mitchell, A.L. Sequence variation in mitochondrial complex I genes: mutation or polymorphism? *J. Med. Genet.* **2005**, *43*, 175–179.

149. Kolarova, H.; Liskova, P.; Tesarova, M.; Kucerova Vidrova, V.; Forgac, M.; Zamecnik, J.; Hansikova, H.; Honzik, T. Unique presentation of LHON/MELAS overlap syndrome caused by m.13046T>C in MTND5. *Ophthalmic Genet.* **2016**, *37*, 419–423.

150. McKenzie, M.; Liolitsa, D.; Akinshina, N.; Campanella, M.; Sisodiya, S.; Hargreaves, I.; Nirmalananthan, N.; Sweeney, M.G.; Abou-Sleiman, P.M.; Wood, N.W.; et al. Mitochondrial ND5 Gene Variation Associated with Encephalomyopathy and Mitochondrial ATP Consumption. *J. Biol. Chem.* **2007**, *282*, 36845–36852.

151. Santorelli, F.M.; Tanji, K.; Kulikova, R.; Shanske, S.; Vilarinho, L.; Hays, A.P.; DiMauro, S. Identification of a Novel Mutation in the mtDNA ND5 Gene Associated with MELAS. *Biochem. Biophys. Res. Commun.* **1997**, *238*, 326–328.

152. Tranah, G.J.; Maglione, J.E.; Yaffe, K.; Katzman, S.M.; Manini, T.M.; Kritchevsky, S.; Newman, A.B.; Harris, T.B.; Cummings, S.R. Mitochondrial DNA m.13514G>A heteroplasmy is associated with depressive symptoms in the elderly. *Int. J. Geriatr. Psychiatry* **2018**, *33*, 1319–1326.

153. Batandier, C.; Picard, A.; Tessier, N.; Lunardi, J. Identification of a novel T398A mutation in the ND5 subunit of the mitochondrial complex I and of three novel mtDNA polymorphisms in two patients presenting ocular symptoms. *Hum. Mutat.* **2000**, *16*, 532–532.

154. Choi, B.-O.; Hwang, J.H.; Kim, J.; Cho, E.M.; Cho, S.Y.; Hwang, S.J.; Lee, H.W.; Kim, S.J.; Chung, K.W. A MELAS syndrome family harboring two mutations in mitochondrial genome. *Exp. Mol. Med.* **2008**, *40*, 354.

155. Tzen, C.-Y.; Thajeb, P.; Wu, T.-Y.; Chen, S.-C. Melas with point mutations involving tRNALeu (A3243G) and tRNAGlu(A14693g). *Muscle Nerve* **2003**, *28*, 575–581.

156. Ravn, K.; Wibrand, F.; Hansen, F.J.; Horn, N.; Rosenberg, T.; Schwartz, M. An mtDNA mutation, 14453G→A, in the NADH dehydrogenase subunit 6 associated with severe MELAS syndrome. *Eur. J. Hum. Genet.* **2001**, *9*, 805–809.

157. De Coo, I.F.M.; Renier, W.O.; Ruitenbeek, W.; Ter Laak, H.J.; Bakker, M.; Sch�gger, H.; Van Oost, B.A.; Smeets, H.J.M. A 4-base pair deletion in the mitochondrial cytochromeb gene associated with parkinsonism/MELAS overlap syndrome. *Ann. Neurol.* **1999**, *45*, 130–133.

158. Emmanuele, V.; Sotiriou, E.; Rios, P.G.; Ganesh, J.; Ichord, R.; Foley, A.R.; Akman, H.O.; DiMauro, S. A Novel Mutation in the Mitochondrial DNA Cytochrome b Gene (MTCYB) in a Patient With Mitochondrial Encephalomyopathy, Lactic Acidosis, and Strokelike Episodes Syndrome. *J. Child Neurol.* **2013**, *28*, 236–242.

159. Mancuso, M.; Nesti, C.; Ienco, E.C.; Orsucci, D.; Pizzanelli, C.; Chiti, A.; Giorgi, F.S.; Meschini, M.C.; Fontanini, G.; Santorelli, F.M.; et al. Novel MTCYB mutation in a young patient with recurrent stroke-like episodes and status epilepticus. *Am. J. Med. Genet. Part A* **2014**, *164*, 2922–2925.

160. Gil Borlado, M.C.; Moreno Lastres, D.; Gonzalez Hoyuela, M.; Moran, M.; Blazquez, A.; Pello, R.; Marin Buera, L.; Gabaldon, T.; Garcia Peñas, J.J.; Martín, M.A.; et al. Impact of the Mitochondrial Genetic Background in Complex III Deficiency. *PLoS One* **2010**, *5*, e12801.

161. Sato, T.; Muroya, K.; Hanakawa, J.; Iwano, R.; Asakura, Y.; Tanaka, Y.; Murayama, K.; Ohtake, A.; Hasegawa, T.; Adachi, M. Clinical manifestations and enzymatic activities of mitochondrial respiratory chain complexes in Pearson marrow-pancreas syndrome with 3-methylglutaconic aciduria: a case report and literature review. *Eur. J. Pediatr.* **2015**, *174*, 1593–1602.

162. Williams, T.B.; Daniels, M.; Puthenveetil, G.; Chang, R.; Wang, R.Y.; Abdenur, J.E. Pearson syndrome: Unique endocrine manifestations including Neonatal Diabetes and adrenal insufficiency. *Mol. Genet. Metab.* **2012**, *106*, 104–107.

163. Rötig, A.; Bourgeron, T.; Chretien, D.; Rustin, P.; Munnich, A. Spectrum of mitochondrial DNA rearrangements in the Pearson marrow-pancreas syndrome. *Hum. Mol. Genet.* **1995**, *4*, 1327–1330.

164. Kapsa, R.; Thompson, G.N.; Thorburn, D.R.; Dahl, H.-H.M.; Marzuki, S.; Byrne, E.; Blok, R.B. A novel mtDNA deletion in an infant with Pearson syndrome. *J. Inherit. Metab. Dis.* **1994**, *17*, 521–526.

165. Moraes, C.T.; DiMauro, S.; Zeviani, M.; Lombes, A.; Shanske, S.; Miranda, A.F.; Nakase, H.; Bonilla, E.; Werneck, L.C.; Servidei, S.; et al. Mitochondrial DNA Deletions in Progressive External Ophthalmoplegia and Kearns-Sayre Syndrome. *N. Engl. J. Med.* **1989**, *320*, 1293–1299.

166. Yu, N.; Zhang, Y.; Zhang, K.; Xie, Y.; Lin, X.; Di, Q. MELAS and Kearns–Sayre overlap syndrome due to the mtDNA m. A3243G mutation and large-scale mtDNA deletions. *eNeurologicalSci* **2016**, *4*, 15–18.

167. Hellebrekers, D.M.E.I.; Blakely, E.L.; Hendrickx, A.T.M.; Hardy, S.A.; Hopton, S.; Falkous, G.; de Coo, I.F.M.; Smeets, H.J.M.; van der Beek, N.M.E.; Taylor, R.W. A novel mitochondrial m.4414T>C MT-TM gene variant causing progressive external ophthalmoplegia and myopathy. *Neuromuscul. Disord.* **2019**, *29*, 693–697.

168. Schlapakow, E.; Peeva, V.; Zsurka, G.; Jeub, M.; Wabbels, B.; Kornblum, C.; Kunz, W.S. Distinct segregation of the pathogenic m.5667G>A mitochondrial tRNAAsn mutation in extraocular and skeletal muscle in chronic progressive external

ophthalmoplegia. *Neuromuscul. Disord.* **2019**, *29*, 358–367.

169. KARADIMAS, C.; SALVIATI, L.; SACCONI, S.; CHRONOPOULOU, P.; SHANSKE, S.; BONILLA, E.; DEVIVO, D.; DIMAURO, S. Mitochondrial myopathy and ophthalmoplegia in a sporadic patient with the G12315A mutation in mitochondrial DNA. *Neuromuscul. Disord.* **2002**, *12*, 865–868.

170. Mordel, P.; Schaeffer, S.; Dupas, Q.; Laville, M.-A.; Gérard, M.; Chapon, F.; Allouche, S. A 2 bp deletion in the mitochondrial ATP 6 gene responsible for the NARP (neuropathy, ataxia, and retinitis pigmentosa) syndrome. *Biochem. Biophys. Res. Commun.* **2017**, *494*, 133–137.

171. Lopez-Gallardo, E.; Solano, A.; Herrero-Martin, M.D.; Martinez-Romero, I.; Castano-Perez, M.D.; Andreu, A.L.; Herrera, A.; Lopez-Perez, M.J.; Ruiz-Pesini, E.; Montoya, J. NARP syndrome in a patient harbouring an insertion in the MT-ATP6 gene that results in a truncated protein. *J. Med. Genet.* **2008**, *46*, 64–67.

172. Donovan, L.E.; Severin, N.E. Maternally Inherited Diabetes and Deafness in a North American Kindred: Tips for Making the Diagnosis and Review of Unique Management Issues. *J. Clin. Endocrinol. Metab.* **2006**, *91*, 4737–4742.

173. Naveed, A.K.; Wahid, M.; Naveed, A. Mitochondrial tRNALeu(UUR) gene mutation and maternally inherited diabetes mellitus in Pakistani population. *Int. J. Diabetes Mellit.* **2009**, *1*, 11–15.

174. Chen, F.L.; Liu, Y.; Song, X.Y.; Hu, H.Y.; Xu, H.B.; Zhang, X.M.; Shi, J.H.; Hu, J.; Shen, Y.; Lu, B.; et al. A novel mitochondrial DNA missense mutation at G3421A in a family with maternally inherited diabetes and deafness. *Mutat. Res. Mol. Mech. Mutagen.* **2006**, *602*, 26–33.

175. Tabebi, M.; Charfi, N.; Kallabi, F.; Alila-Fersi, O.; Ben Mahmoud, A.; Tlili, A.; Keskes-Ammar, L.; Kamoun, H.; Abid, M.; Mnif, M.; et al. Whole mitochondrial genome screening of a family with maternally inherited diabetes and deafness (MIDD) associated with retinopathy: A putative haplotype associated to MIDD and a novel MT-CO2 m.8241T>G mutation. *J. Diabetes Complications* **2017**, *31*, 253–259.
